# Ambrafuran (Ambrox^TM^) Synthesis from Natural Plant Product Precursors

**DOI:** 10.3390/molecules25173851

**Published:** 2020-08-25

**Authors:** Efficient N. Ncube, Lucia Steenkamp, Ian A. Dubery

**Affiliations:** 1 Research Centre for Plant Metabolomics, Department of Biochemistry, University of Johannesburg, P.O. Box 524, Auckland Park 2006, South Africa; 201004877@student.uj.ac.za; 2 Chemicals Cluster, Council for Scientific and Industrial Research (CSIR), P.O. Box 395, Pretoria 0001, South Africa; lsteenkamp@csir.co.za

**Keywords:** ambrafuran, fragrance, labdane, synthesis, terpenes, terpenoids

## Abstract

Ambergris, an excretion product of sperm whales, has been a valued agent in the formulation of perfumes. The composition of ambergris consists of two major components: 40–46% cholestanol type steroids and approximately 25–45% of a triterpenoid known as ambrein. Ambergris undergoes oxidative decomposition in the environment to result in odorous compounds, such as ambraoxide, methylambraoxide, and ambracetal. Its oxidized form, ambrafuran (IUPAC name: 3a,6,6,9a-tetramethyl-2,4,5,5a,7,8,9,9b-octahydro-1*H*-benzo[e][1]benzofuran), is a terpene furan with a pleasant odor and unique olfactive and fixative properties. The current state of the fragrance industry uses ambrafuran materials entirely from synthetic or semisynthetic sources. However, natural compounds with the potential to be converted to ambergris-like odorants have been extracted from several different types of plants. Here we review plant terpenoids suitable as starting materials for the semisyntheses of ambrafuran or intermediates, such as ambradiol, that can be used in biocatalytic transformations to yield ambrafuran.

## 1. Ambrafuran, a High Value, Fine Fragrant Perfume Ingredient

**Figure 1:**
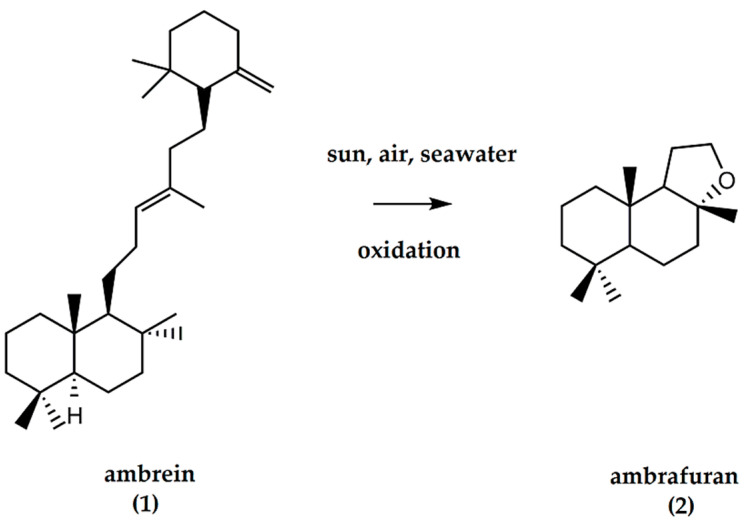
Ambrein (C_30_H_52_O), a constituent of ambergris and precursor of (±)-ambrafuran (C_16_H_28_O). Ambergris, a derivatized triterpenoid / naphthol compound, undergoes oxidative decomposition by the action of seawater, air, and sunlight to generate ambrafuran, a norlabdane tricyclic ether.

Historically, ambrafuran was obtained from ambergris, a waxy excretion product from sperm whales (*Physeter macrocephalus* L.) [1]. Ambergris has a “subtle odor reminiscent of seaweed, wood and moss, but with a peculiar sweet, yet dry undertone of unequalled tenacity” [2,3]. The chemical components within ambergris include a substituted triterpenoid known as ambrein (**1**). Due to environmental exposure and the action of seawater, air, sunlight, etc., ambergris undergoes oxidative decomposition to generate odorous degradation compounds known as ambroxides (Figure 1). Some of the degradative routes have been chemically simulated, and due to the endangered status of the sperm whale, synthetic ambroxides have now replaced ambergris in the manufacture of perfume [4]. Although some of the chemical pathways developed for the degradation of ambergris generate odorless products, most of these have resulted in products exhibiting odors reminiscent of musk [5]. Among the most interesting ambergris fragrance compounds is ambrafuran (**2**) or 3a,6,6,9a-tetramethyl-2,4,5,5a,7,8,9,9b-octahydro-1H-benzo[e][1]benzofuran, regarded as the prototype of all ambergris odorants. Synonyms include ambroxide (Ambrox^TM^)/ambraoxide, methylambraoxide, ambracetal, amberlyn, ambronide, and (−)-norlabdane oxide [6]. (−)-Ambrafuran has emerged as the leading and most relevant among the ambergris compounds, owing to the delicate odor, an animalic note characteristic of the material, and its fixative properties [4,7]. Accordingly, it is considered as a high value, fine fragrant ingredient. Interestingly, (−)-ambrafuran presents a much stronger odor than its (+)-ambrafuran antipode with a somewhat different odor [8,9].

A number of synthetic routes to (−)-ambrafuran have been described in the literature from naturally-occurring terpenoids [1,10,11]. Following the lapse of patents for the production of (−)-ambrafuran in the 1980s, a highly competitive race followed to research structurally related natural products from which (−)-ambrafuran could be chemically synthesized. By the early 1990s, totally synthetic versions of racemic (±)-ambrafuran diastereoisomers were introduced to replace ambrafuran derived from ambergris. This replacement was considered as a substitute to relieve the burden on natural resources, which might either be unreliable and unavailable or lead to the production of impure compounds [12].

Regarding the synthesis of the racemate, (±)-ambrafuran, various complete synthetic routes have been reviewed and developed on the basis of biogenetic-type cyclizations from farnesoic acid, monocyclofarnesoic acid, and derivatives thereof [13]. Versions of these synthetic racemic ambrafuran diastereomers have been introduced under various trade names [14]. However, some of these syntheses failed to be industrially feasible or commercially viable [15]. 

Labdane is a natural bicyclic diterpene that forms the structural core for a wide variety of natural products. Since (−)-ambrafuran is a tetranorlabdane diterpenoid, the labdane diterpenoids are regarded as the most ideal (albeit not the only) starting material for its production [1,4,11]. Accordingly, this review classifies the known syntheses of (−)-ambrafuran into three classes based on their unique starting materials, namely (i) synthesis starting from cyclic monoterpenoids, (ii) synthesis starting from sesquiterpenoids, and (iii) synthesis starting from diterpenes [16]. Below follows a broad description of published procedures for the synthesis of pure ambrafuran. Details about the catalysts and chemical procedures used in these syntheses, as well as yield, racemization, and diastereoisomeric excess, can be found in the original cited sources.

## 2. Synthesis Starting from Monoterpenes

### 2.1. (+)-Carvone

(+)-Carvone is a monocyclic terpene and dominant constituent of seed oils of caraway (*Carum carvi*) and dill (*Anethum graveolens*), as well as leaves of members of the Lamiaceae family. Carvone is economically available in both enantiomerically pure forms, making it an attractive starting material for the asymmetric total synthesis of natural products such as ambrafuran [17,18].

**Figure 2:**
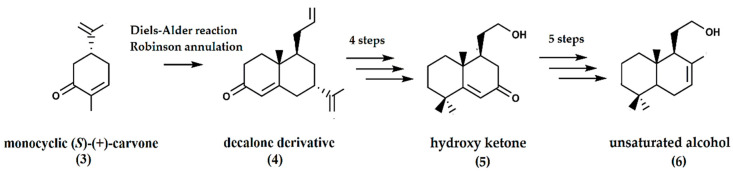
Synthesis of ambrafuran from monocyclic S-(+)-carvone (C_10_H_14_O). Adapted from Verstegen-Haaksma et al. [17].

From monocyclic (*S*)-(+)-carvone (**3**), bicyclic intermediates such as decalone (**4**) were synthesized from carvone using two annulation methodologies, i.e., the Diels–Alder reaction and the Robinson annulation [17]. The conversion of the decalone derivative to a hydroxy ketone (**5**) involved the conversion of the allyl moiety into a hydroxy ethylene moiety and the conversion of the isopropenyl group into carbonyl functionality. In the subsequent steps, a methyl group and double bond was introduced to result in the unsaturated alcohol (**6**) (Figure 2) that was then cyclized using p-toluenesulfonic acid at room temperature to yield (−)-ambrafuran in a 9% overall yield. 

### 2.2. Thujone

Thujone is a monoterpene ketone. It is found in high concentrations in branches and needles of the *Thuja plicata* (Western red cedar) tree as well as a number of plants, such as arborvitae (genus Thuja), Nootka cypress, some junipers, mugwort, oregano, common sage, tansy, mint, and wormwood. The compound is often obtained as a waste product from the Canadian forest industries [19]. It occurs naturally in two diastereomeric forms: (−)-α-thujone and (+)-β-thujone, usually in an isomer ratio of 1:2. In addition, both epimers are present in variable amounts in a number of crude essential oils [20].

**Figure 3:**
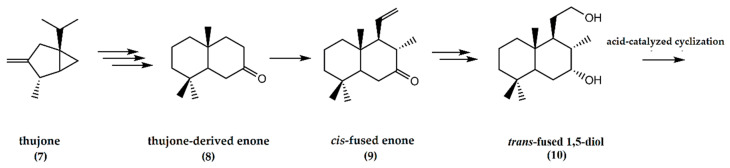
Synthesis of ambrafuran from (+)-beta-thujone, C_10_H_16_O, a monoterpene ketone. Adapted from Kutney and Chen [19].

The initial conversion of thujone (**7**) to yield the thujone-derived enone (**8**) involved seven steps. The subsequent steps in the chemical synthetic pathway, as summarized in Figure 3, involve the conversion of the enone to a *cis*-fused enone (**9**). The final steps involve the acid-catalyzed cyclization of the *trans*-fused 1,5-diol (**10**) to afford the tetrahydrofuran ring system present in ambrafuran [19].

## 3. Synthesis Starting from Sesquiterpenoids

### 3.1. Beta-Ionone

Beta-ionone (part of a series of closely related chemical substances) is derived from the degradation of plant carotenoids and is a common aroma compound found in a variety of essential oils. As such, it is an important fragrance chemical used in perfumery [3].

**Figure 4:**
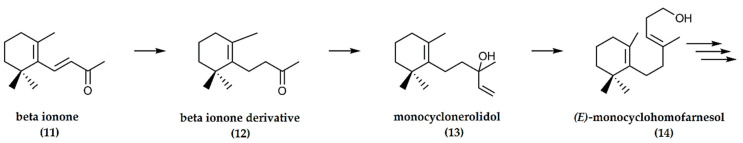
Synthesis of ambrafuran from β-ionone, a C_13_H_20_O sesquiterpenoid. Adapted from Barrero et al. [13].

In the synthesis of ambrafuran from β-ionone (**11**) as per Figure 4, the first step involved selective hydrogenation resulting in the formation of a β-ionone derivative (**12**). Subsequently, the derivative was converted to monocyclonerolidol (**13**) followed by *(E)*-monocyclohomofarnesol (**14**). The final steps involved multiple reactions that finally resulted in the synthesis of racemic ambrafuran [13]. 

### 3.2. Nerolidol

Nerolidol is a naturally occurring acyclic sesquiterpene alcohol, found in the essential oils of many types of plants and flowers. There are two isomers of nerolidol, *cis* and *trans*, which differ in the geometry about the central double bond. It is used as a flavoring agent and in perfumery with a woody/fresh bark aroma [21].

**Figure 5:**
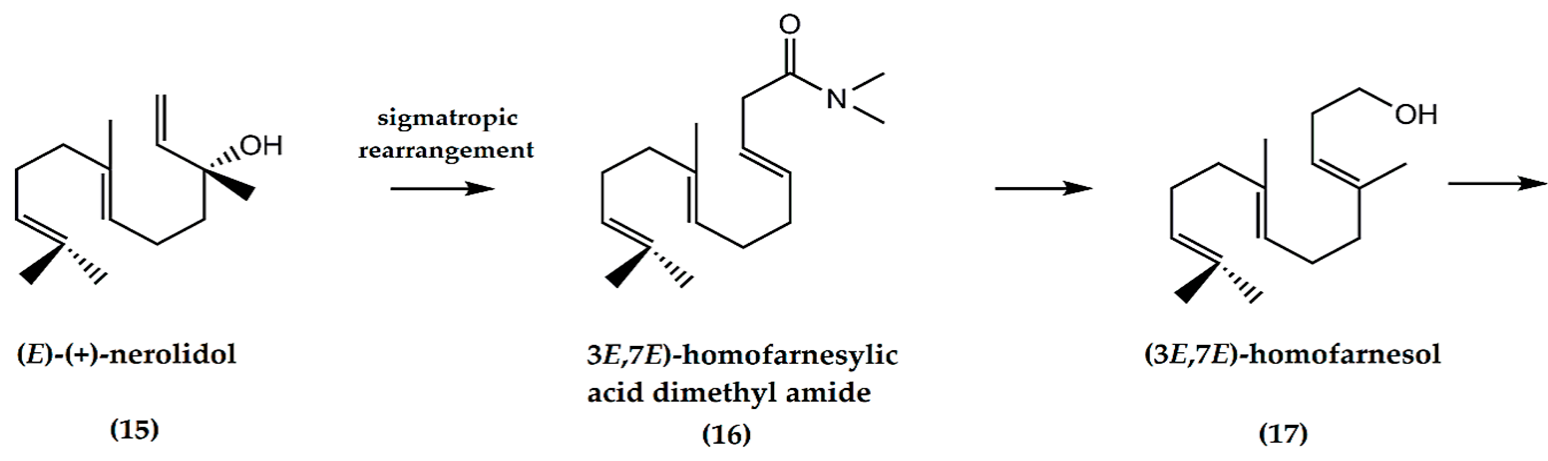
Synthesis of ambrafuran from nerolidol (C_15_H_26_O). Adapted from Aoki and Ataka [22].

A three-step synthesis of racemic (±) ambrafuran from (*E*)-(+)-nerolidol (**15**) (Figure 5) was first reported by Barrero et al. [13]. The crucial step involves the sigmatropic rearrangement of an allylic alcohol to the homologous (3*E*,7*E*)-homofarnesylic acid dimethyl amide (**16**) using *N*,*N*-dimethyl formamide dimethyl acetal. The amide is then converted to (3*E*,7*E*)-homofarnesol (**17**) leading to the formation of ambrafuran. The main advantage of this route is the small number of steps required relative to other syntheses [22].

### 3.3. β-Farnesene

Farnesene is a term generally used to describe six different types of sesquiterpenes with similar chemical properties. These sesquiterpenes are categorized into two major classes, i.e., α and β, both of which are naturally occurring in a variety of different plants. The β isomer, chemically known as (7,11-dimethyl-3-methylene-1,6,10-dodecatriene), exists as two stereoisomers distinguished by the geometry of its central double bond. The *E* isomer is a constituent in various essential oils, characterized by a woody odor [23,24]. Noteworthy is the production of diastereoisomeric intermediates, particularly for the synthesis of (±) ambrafuran from the farnesene backbone and derivatives, as demonstrated in the literature [14,23,25,26]. Fortunately, these diastereoisomers can be chromatographically separated with ease [23].

**Figure 6:**
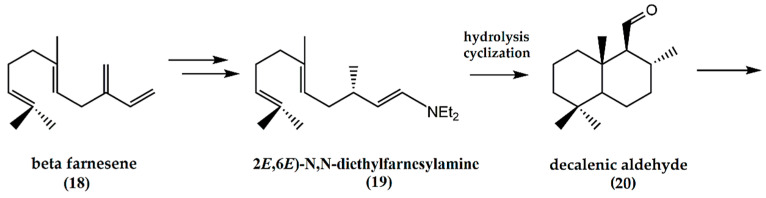
Schematic representation of synthesis of ambrafuran from β-farnesene (C_15_H_24_). Adapted from Serra [26].

The synthesis of (−)-ambrafuran from the acyclic β-farnesene (**18**) (Figure 6) has been reported [27]. (*E*)-β-farnesene is converted to *N*,*N*-diethylfarnesylenamine in a two-step reaction with diethylamine to afford (2*E*,6*E*)-*N,N*-diethylfarnesylamine (**19**). Through hydrolysis and cyclization of the enamine, formation of a decalenic aldehyde (**20**) resulted, which can be used as a chiral building block for (−)-ambrafuran synthesis as illustrated above.

### 3.4. Drimenol

(−)-Drimenol is a primary alcohol containing sesquiterpenoid, chemically an octahydronaphthalene and a homoallylic alcohol. The compound occurs in *Drimys angustifolia* and related species of woody evergreen flowering plants. Members of the family generally have aromatic bark and leaves, and some are used to extract essential oils [28].

**Figure 7:**
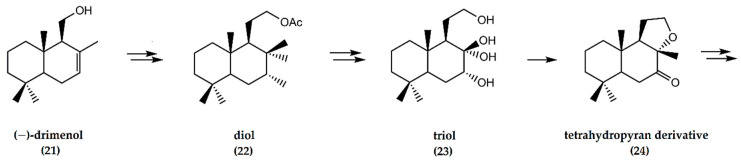
Schematic representation of synthesis of (−)-ambrafuran from (−)-drimenol, a C_15_H_26_O sesquiterpenoid. Adapted from González-Sierra et al. [29].

In the synthesis of ambrafuran, (−)-drimenol (**21**) is initially converted to a diol (**22**) via several chemical steps. Subsequently, the diol undergoes saponification to afford a triol (**23**). Transformation of the triol furnished a tetrahydropyran derivative (**24**) (Figure 7) that, in turn, was oxidized before reduction to yield the desired ambrafuran with an overall yield of 19% [29].

## 4. Synthesis Starting from Diterpenes: The Bicyclic Labdanes

**Figure 8:**
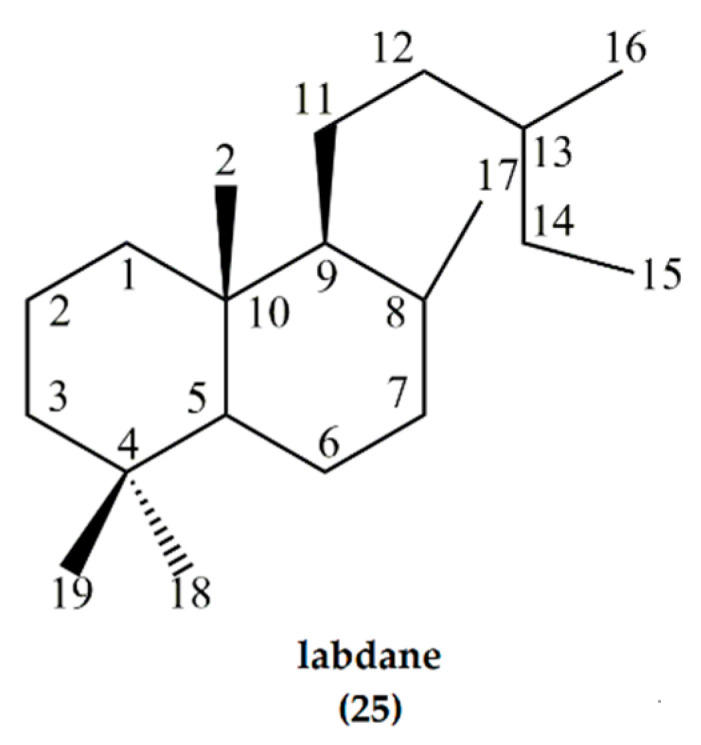
Labdane, a C_20_H_38_ diterpene with a decalin backbone and alkyl substituent. It forms the core structure of a wide variety of natural products collectively known as labdane diterpenes.

Labdane (**25**) is a natural bicyclic diterpene forming the structural core of labdane diterpene natural products [30]. The term “labdane” refers to the saturated hydrocarbon that is structurally characterized by a 4,4,10-trimethyl substituted *trans*-decalin system with a β-oriented substituted alkyl side chain at C-9 (Figure 8). Labdanes such as larixol, manool, and sclareol carry a (3-hydroxy-3-methyl-4-pentenyl)-side chain at C9 [11] while *cis*-abienol and communic acids have a 1,3-diene side chain.

Research has revealed the relationship between the labdane structure and the ambergris scent. Complex natural products can often be synthesized from congeners that occur abundantly in nature. In the case of the labdanes, only a limited number are obtainable in large amounts from plant products, thus limiting their use as starting materials. However, oleoresin obtained from conifers is a rich source of labdanes, important secondary metabolites that are synthesized by diterpene synthases and cytochrome P450s [30]. Due to the importance of ambrafuran in the fragrance industry, the chemistry of many labdanes has been comprehensively investigated, mostly for this purpose [16]. The most well-documented labdanes include labdanolic acids [16] and communic acids [31]. In addition, sclareol, a diterpene labdane from clary sage, has also found great utility in the industrial production of ambrafuran [32]. The basic labdane skeleton consists of a decalin (decahydronaphthalene) backbone with an alcohol, ether or ester moiety, as well as alkyl substituents at specific positions, laying a foundation for all ambergris odorants [33].

### 4.1. Levopimaric Acid 

(−)-Levopimaric acid is a dominant abietane-type (C_20_H_30_O_2_, with two double bonds in the molecule) diterpene acid (**26**) extracted from the acid portion of conifer oleoresin, a major component of conifer trees. Following conversion to the methyl derivative, oxidative cleavage of ring C is facilitated by the C-4 chiral carbon belonging to (−)-levopimaric acid that serves as a perfect synthon for the reduction of the carboxylate function to a methyl group and for the (−)-ambrafuran skeleton [34].

**Figure 9:**
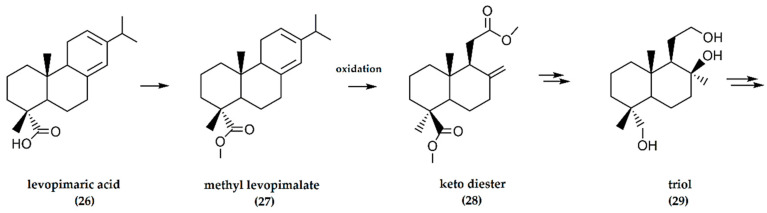
Synthesis of (−)-ambrafuran from (−)-levopimarate (C_20_H_30_O_2_). Adapted from Yasutaka and Kiroichi [34].

In this synthetic pathway, the carboxylic acid function at C-4 is methylated, and the methyl levopimalate (**27**) is oxidized by Pelletier’s method to a keto diester (**28**), followed by Wittig olefination of the keto moiety. Intermediate products include an exocyclic methylene and epoxide, followed by a reduction of the α-epoxide to a triol (**29**) (Figure 9). Mesylation of the hydroxymethyl group at C-4 and nucleophilic ring-closure afforded the tricyclic mesylate, the reduction of which gave a 44% yield of (−)-ambrafuran [34].

### 4.2. Manoyl Oxide 

Manoyl oxide is a diterpene (labd-14-ene, 8,13-epoxy-, (13S)-epimanoyl oxide) present in the oleoresin and essential oil of needles of several pine trees such as *Pinus resinosa, P. sylvestris,* and the Siberian fir tree, *Abies sibirica* [35].

**Figure 10:**
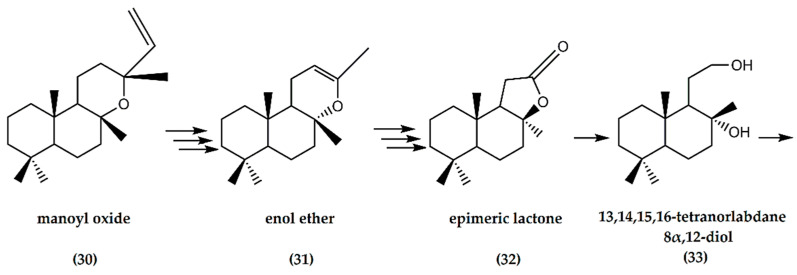
Schematic representation of synthesis of (−)-ambrafuran-derivative (8a,12-epoxy-13,14,15,16-tetranorlabdane) from (−)-manoyl oxide (C_20_H_34_O). Adapted from Cambie et al. [36].

Regarding the chemical synthetic pathway of (−)-ambrafuran, the (−)-manoyl oxide (**30**) is converted to an enol ether (**31**) in a multi-step reaction. Through multiple oxidation steps of the enol ether, a corresponding epimeric lactone (**32**) was obtained, which, in turn, resulted in the formation of a 13,14,15,16-tetranorlabdane 8α,12-diol (**33**), Figure 10. The final step involved a dehydration reaction, where concentration of the dried extract resulted in 8α,12-epoxy-13,14,15,16-tetranorlabdane (overall yield of 17%) with an ambergris-type odor [36]. In addition, García-Granados et al. [37] showed the semisynthesis of 3-hydroxyderivatives of ambrafuran from the starting material of ent-3,12a-dihydroxy-13-epi-manoyl oxide, by combining chemical and biotransformation procedures.

### 4.3. Abietic Acid 

(−)-Abietic acid, a C_20_H_30_O_2_ abietane diterpenoid, that is an abieta-7,13-diene substituted by a carboxy group at position 18 (**34**). Similar to other labdanes, abietic acid is an easily obtainable and abundant constituent of conifer resin. It is the primary component of resin acid, the most abundant of several closely related organic acids that constitute most of rosin, the solid portion of the oleoresin [38].

**Figure 11:**
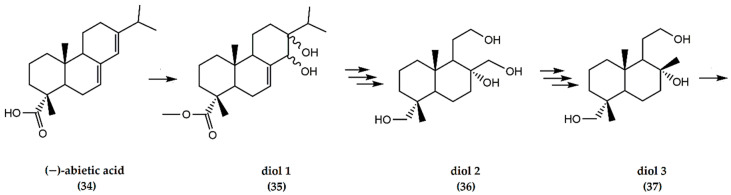
Schematic representation of synthesis of ambrafuran from (−)-abietic acid (C_20_H_30_O_2_). Adapted from Koyama et al. [39].

A multifaceted synthesis of (±) ambrafuran from (−)-abietic acid (Figure 11), involves multi-step reactions to furnish various diol-related intermediate structures (**35**, **36**, **37**) leading to the formation of ambrafuran with an overall yield of 97% from (**37**) [39].

### 4.4. Labdanolic Acid

**Figure 12:**
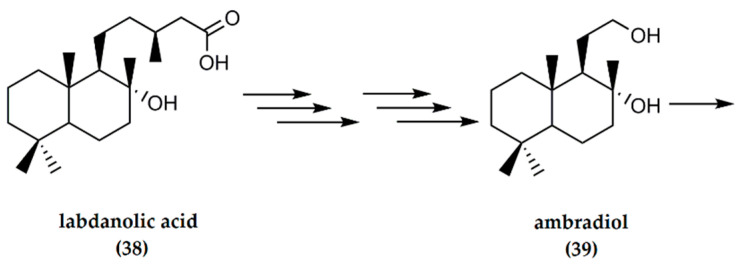
Synthesis of (−)-ambrafuran from labdanolic acid, a (C_20_H_36_O_3_) labdane diterpenoid. Adapted from Bolster [16].

Labdanolic acid is a natural product extracted from *Cistus ladaniferus* and *C. creticus* L., species of “rock-rose” [9]. The raw resin is usually extracted by boiling the leaves and twigs of the plants to generate gum labdanum. Labdanolic acid has the potential to serve as an inexpensive starting material for the synthesis of ambrafuran, since, after oxidative degradation of the C-9 side chain, suitable synthons for (−)-ambrafuran synthesis are generated [16]. (−)-Ambrafuran can be obtained from labdanolic acid (**38**) in seven steps, as indicated in Figure 12 below. Initially, labdanolic acid undergoes oxidative degradation in a total of six steps to result in ambradiol (**39**), although in relatively small yields. The intermediate products include triols, cyclic enol ethers, and methyl ketones. Ambradiol is ultimately converted to (−)-ambrafuran (87% yield) the presence of p-toluenesulfonic acid [16].

### 4.5. Cis-Abienol

*Cis*-abienol is a constituent of *P. strobus* and can also be isolated from Canadian balsam, the oleoresin of *Abies balsamea* [16]. It is a labdane diterpenoid containing a tertiary alcohol (**40**). The labdane skeleton has double bonds at C-12 (with Z-stereochemistry) and C-14 and carries a hydroxy group at position C-8. The structural features (*trans*-decalin junction, β-side chain, and a diene system prone to cleavage of the C(12)-C(13) bond) converts *cis*-abienol to a good chiral synthon and starting material for the synthesis of (−)-ambrafuran [13].

**Figure 13:**
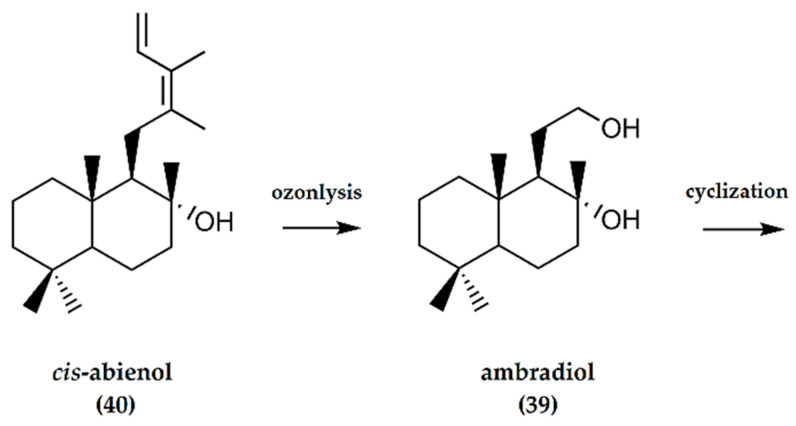
Schematic representation of synthesis of ambrafuran from *cis*-abienol, a C_20_H_34_O labdane diterpenoid. Adapted from Barrero et al. [40].

A two-step synthesis of (−)-ambrafuran using +(-)*cis*-abienol (**40**) as a starting material (Figure 13), first reported by Barrero et al. [40], involves the ozonolysis of +(-)*cis*-abienol to furnish ambradiol (**39**). The diol is then converted to produce (−)-ambrafuran using tosyl chloride in pyridine.

### 4.6. Communic Acids

Communic acids, both *trans*- and *cis*-isomers, are labdane diterpenes naturally synthesized in different species of the Cupressaceae family, such as *Juniperus sabina* [31]. These compounds have a carboxylic acid moiety at C-4, an exocyclic methylene at C-8 and a 1,3-diene side chain at C-9 (**41**) (Figure 8). These materials are used to produce perfume fixatives [31].

**Figure 14:**
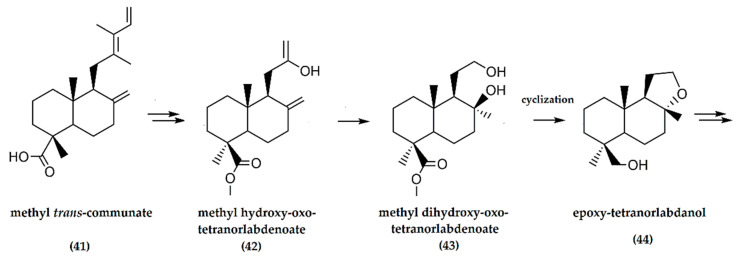
Schematic representation of synthesis of (−)-ambrafuran from methyl *trans*-communate, a C_20_H_30_O_2_ labdane diterpenoid. Adapted from Barrero et al. [41].

The work by Barrero et al. [41] reported the synthesis of ambrafuran from communic acids via ozonolysis and oxidation to methyl hydroxy-oxo-tetranorlabdenoate (**42**), conversion to methyl dihydroxy-oxo-tetranor-labdenoate (**43**) which was further cyclized to produce the alcohol epoxy-tetranorlabdanol (**44**) (Figure 14). The final steps involved the transformation of epoxy-tetranorlabdanol to (−)-ambrafuran with an overall yield of 71%. 

### 4.7. Sclareol

**Figure 15:**
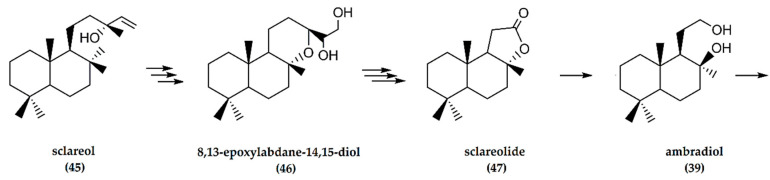
Schematic representation of synthesis of (−)-ambrafuran from sclareol (C_20_H_36_O_2_). Adapted from Hinder and Stoll [46].

Sclareol is a diterpene natural product that does not have a wide distribution in plants but is nonetheless the most used labdane in industry related to (−)-ambrafuran production [12,32]. Sclareol is extracted from leaves of *Nicotiana glutinosa* and, more frequently, the flowers and leaves of the biennial herb *Salvia sclarea* (clary sage), a member of the Lamiaceae family [42] native to Southern Europe, the Mediterranean basin, and Iran [5]. It is a fragrant chemical with a sweet balsamic scent but low odor strength and has been investigated for antitumor, antifungal, and antibacterial properties [43]. Sclareol (**45**) consists of a labdane carbon skeleton with a hydroxyl group at C-8 (Figure 15), resembling that of (−)-ambrafuran. This structural similarity has made sclareol the most promising starting material for semisynthesis production of ambrafuran [5,44]. The main industrial extraction procedure to isolate sclareol from clary sage involves hydro-distillation of the aerial parts of the plant, followed by solvent extraction of the remaining plant material [45].

The chemical synthesis of either ambradiol (tetranorlabdane diol) or sclareolide—both important precursors for ambrafuran production from sclareol—involves a seven-step oxidative degradation (Figure 15) [47]. In its first implementation, the terminal alkene of sclareol (**45**) was dihydroxylated, followed by oxidative cleavage of the resultant triol [48]. To avoid the need to constantly recover ruthenium salts, a process using epoxidation of sclareol followed by Payne rearrangement and acid cyclization yielded 8,13-epoxylabdane-14,15-diol (**46**) in solid form. Periodate cleavage of the diol gave the 8,13-epoxy-15-norlabdane-14-aldehyde, followed by Jones oxidation to produce sclareolide (**47**) without traces of acetoxy acid. Reduction of the lactone with lithium aluminiumhydride yielded ambradiol (**39**), considered the most suitable intermediate compound for ambrafuran production [47]. Cyclodehydration of ambradiol at room temperature formed the furan over activated zeolite in hexane or toluene to yield (−)-ambrafuran [49].

Much research has been dedicated to improving the chemical synthesis described above, as evidenced by the number of reports focused on the synthesis of ambrafuran from sclareol [6,40,50,51,52]. Frequently, the synthesis of ambrafuran seemingly results in racemic mixtures rather than distinct diastereoisomers. As a result, Farbood et al. [53] developed and patented a biotechnological route using bacteria to convert racemic sclareol to the enantiomeric pure intermediate, (−)-ambradiol. 

## 5. Conclusions and Perspectives

Ambrafuran is the leading fragrance compound owing to its fixative properties and animalic fragrance note characteristic [14]. The demand for the internationally valued compound has resulted in the development of several pathways for chemical synthesis, as highlighted in this review. Aspects related to the yield and purity of the end product as well as possible detrimental environmental effects (of solvents, catalysts, and byproducts) of the whole process should be factors to consider in the choice of starting material and synthetic route(s) [54]. Here, a focus on green chemistry necessitates that the design of chemical products and processes that reduce or eliminate the use or generation of hazardous substances be considered. As such, plant-based starting materials containing the labdane skeleton are desirable raw materials for the semisynthesis of ambrafuran. Although much ground has been covered over the years in this area, there is a need for exploring new avenues for the semisynthetic production of ambrafuran integrated with combinatorial synthetic biology (SynBio) and metabolic engineering approaches. 

Evidently, the use of sclareol is the most established precursor for ambrafuran synthesis [26], and, in order to provide a basis for an alternative route to sclareol, its biosynthetic pathway was successfully reconstructed in genetically engineered *Escherichia coli* [42] for subsequent conversion to ambrafuran. Relatedly, a two-step semisynthetic process for producing ambrafuran starting from sclareol was developed [49]. The filamentous yeast, *Hyphozyma roseoniger (Moesziomyces antarcticus),* was used in the first step to transform sclareol into ambradiol. The obtained ambradiol was subsequently converted to (−)-ambrafuran using a zeolite-based cyclization procedure.

Currently, there is on-going research on biocatalytic routes for ambrafuran and ambergris-like odorants as a substitute for the chemical routes [26,42,55,56]. One such approach is the use of fermentation processes based on synthetic biology and white biotechnology (the use of living cells—from yeast, molds, bacteria, and plants [57]—and enzymes to synthesize ambrafuran that require less energy and create less waste during their production) [58], coupled with proprietary green chemistry technologies [59].
